# Dynamic Characterization of Optical Coherence-Based Displacement-Type Weight Sensor

**DOI:** 10.3390/s23218911

**Published:** 2023-11-02

**Authors:** Zhengchuang Lai, Zhongjie Ouyang, Shuncong Zhong, Wei Liang, Xiaoxiang Yang, Jiewen Lin, Qiukun Zhang, Jinlin Li

**Affiliations:** 1 Institute of Mechanical Engineering and Automation, Fuzhou University, Fuzhou 350108, China; m160210011@fzu.edu.cn (Z.L.); 200227026@fzu.edu.cn (Z.O.); sczhong@fzu.edu.cn (S.Z.); yangxx@fzu.edu.cn (X.Y.); linjw@fzu.edu.cn (J.L.); qk_zhang@fzu.edu.cn (Q.Z.); jimlinlee@fzu.edu.cn (J.L.); 2 Fujian Provincial Key Laboratory of Force Measurement, Fujian Metrology Institute, Fuzhou 350010, China; 3 Institute of Electrical Engineering, Jiangxi Metallurgical Vocational and Technical College, Xinyu 338015, China; 4 Fujian Provincial Key Laboratory of Terahertz Functional Devices and Intelligent Sensing, Fuzhou 350108, China

**Keywords:** optical coherence, weight sensor, displacement type, dynamic characteristics

## Abstract

Dynamic characteristics play a crucial role in evaluating the performance of weight sensors and are essential for achieving fast and accurate weight measurements. This study focuses on a weight sensor based on optical coherence displacement. Using finite element analysis, the sensor was numerically simulated. Frequency domain and time domain dynamic response characteristics were explored through harmonic response analysis and transient dynamic analysis. The superior dynamic performance and reduced conditioning time of the non-contact optical coherence-based displacement weight sensor were confirmed via a negative step response experiment that compared the proposed sensing method to strain sensing. Moreover, dynamic performance metrics for the optical coherence displacement-type weight sensor were determined. Ultimately, the sensor’s dynamic performance was enhanced using the pole-zero placement method, decreasing the overshoot to 4.72% and reducing the response time to 0.0132 s. These enhancements broaden the sensor’s operational bandwidth and amplify its dynamic response capabilities.

## 1. Introduction

Dynamic weight measurement, a pervasive sensing technique that is used in daily life, is employed extensively in areas such as traffic management, chemical and metallurgical sectors, construction monitoring, and industrial and agricultural production. For instance, it addresses vehicle overloading issues on expressways [1,2], measures production in challenging environments such as oil extraction and rare earth exploration [3], ensures the safety of high-voltage transmission lines [4,5], and oversees the quality of industrial and agricultural outputs [6,7]. In these challenging environments characterized by high temperatures, excessive humidity, or strong electromagnetic interference, conventional electrical weight sensors can falter. They are susceptible to performance inconsistency due to electromagnetic interference, and they may pose fire and explosion hazards when weighing inflammable or explosive materials. Emerging as a focal point of research, fiber optic sensing technology offers notable advantages: it is lightweight, highly sensitive, safe, resistant to high temperatures, and impervious to electromagnetic interference [8,9]. Thus, it addresses some of the drawbacks of traditional electrical weight sensors and, thereby, is a promising avenue in weight sensor technology research. For example, Zhao et al. [10] introduced a weight sensor rooted in the fiber Bragg grating (FBG) birefringence for vehicle management. Ma et al. [4] crafted a dual-beam weight sensor using FBG sensing principles for ice monitoring on high-voltage transmission lines. Liu et al. [11] unveiled a Sagnac interferometer-based weight sensor built on optical interference principles, while Kumar et al. [12] presented a single-fiber Mach–Zehnder interferometer-based weight sensor that also hinged on optical interference. Furthermore, our team previously explored a dual-beam weight sensor grounded in optical coherence principles [13]. However, most of these studies mainly concentrate on the static performance of such sensors, often sidelining their dynamic capabilities.

The weighing process is typically real-time and dynamic. Using the traditional approach of “static calibration for dynamic use” might not yield the same accuracy and reliability during dynamic weighing as during static weighing. Thus, examining the dynamic characteristics of weight sensors is of utmost importance. Park et al. [14] studied the dynamic performance of dual-eye six-dimensional force sensors, leveraging both numerical simulations and harmonic excitation experiments. They elucidated the relationship between sensor sensitivity and natural frequencies. Gao Changying et al. [15] crafted a mathematical model for the piezoelectric torque sensor using dynamic theory. They then employed the impact response method to discern the sensor’s natural frequencies. Li et al. [16] utilized finite element analysis to delve into the natural frequencies and mode shapes of a piezoelectric six-dimensional force sensor. Following this, they undertook dynamic characteristic analyses of the sensor using pulse load experiments, gleaning insights into the amplitude–frequency and phase frequency curves of the sensor’s transfer function. Drawing from Kane’s theoretical framework, Yao Jiantao et al. [17] introduced the angular velocity operator matrix. They formulated a dynamic model for a comprehensive, preloaded six-dimensional force sensor. To validate their theoretical model, they used finite element analysis and step response experiments. Nonetheless, most research on sensors rooted in optical sensing principles remains exploratory. There is a paucity in the literature discussing the dynamic characteristics of force sensors that rely on optical principles.

In this paper, the dynamic characteristics of a displacement-type weight sensor based on optical coherence principles are explored. Two methods, harmonic response analysis and transient dynamic analysis, were utilized to investigate the frequency and time domain dynamic response characteristics of the weight sensor. The dynamic performance of weight sensors based on two distinct sensing principles was assessed through a negative step response experiment that was conducted. Subsequently, the dynamic performance indices of the weight sensors were analyzed. By employing system identification, the time domain and frequency domain response curves of the sensors were derived. Finally, the dynamic performance of the weight sensors was enhanced and corrected using the pole-zero placement method.

## 2. Structure and Principles of Optical Coherence-Based Displacement-Type Weight Sensor

### 2.1. Weight Sensor Structure

**Figure 1:**
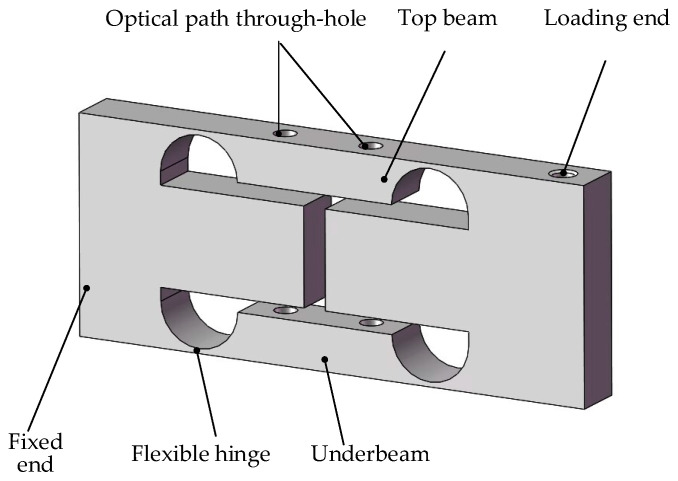
Schematic structure of the double-beam elastomer.

In this study, a dual-beam elastomer structure is proposed by enhancing the traditional double-hole parallel beam, as illustrated in Figure 1. This structure not only retains the benefits of the traditional double-hole parallel beam elastomer but also incorporates a flexible hinge structure to boost the overall performance of the sensor.

**Figure 2:**
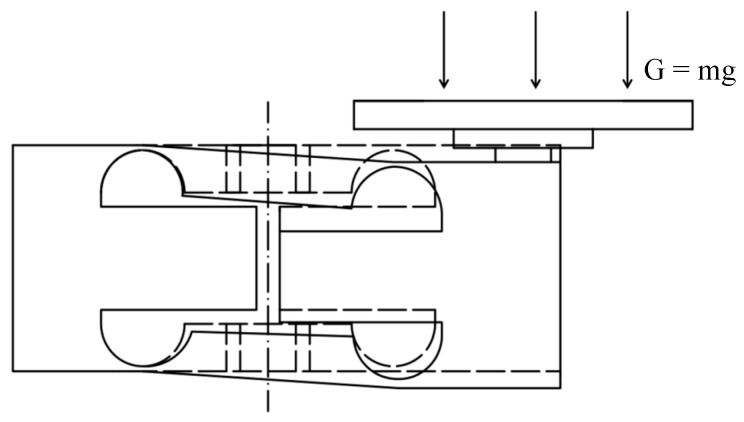
Comparison of elastomer deformation under load.

When subjected to an external load, the dual-beam elastomer’s flexible hinges undergo deformation, leading to the elastomer’s deformation, as depicted in Figure 2. The load passes through these hinges, amplifying the dual-arm’s deformation and thereby enhancing sensor sensitivity. Due to the complementary deformation of the flexible hinges, the dual arms move in parallel, ensuring a linear sensor output. Furthermore, when facing bending moments, the interactions of the four hinges effectively neutralize each other, bolstering the sensor’s resilience to off-center loads. The elastomer features both a reference arm and a measuring arm, facilitating non-contact displacement sensing via optical coherence principles and consequently minimizing sensor hysteresis errors. This design utilizes the differential measurement principle, capturing the displacement variance between the measuring and reference arms, which in turn diminishes the effects of external vibrations on elastomer displacement measurements.

### 2.2. Principles of Optical Coherence-Based Displacement-Type Weight Sensing

**Figure 3:**
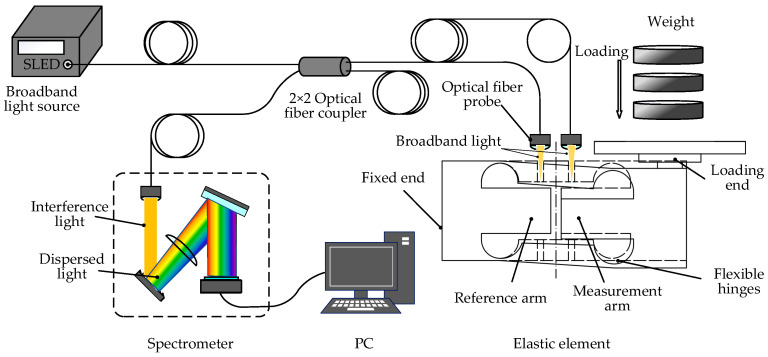
Optical coherence-based displacement-type weight sensing model.

The optically coherent displacement weighing sensing method is an interferometric displacement weighing sensing method based on the principle of low-coherence optical interference, which is proposed by combining the improvement of the traditional Michelson interferometer with the principle of spectral interference. The specific weighing sensing model is shown in Figure 3. The optical coherent displacement weighing sensing model is composed of five core parts: broadband light source, Michelson interferometer, spectrometer, elastomer and computer.

The deformation and displacement of the elastic element were measured using a custom optical coherent system. This system uses a superluminescent diode as the light source and a 2 × 2 fiber coupler. The low-coherent light emitted by the coupler is divided into two interferometric-capable beams; one beam is focused on the reference arm, and the other beam is focused on the measuring arm. The light reflected and scattered from the reference and measuring arms interfered with the 2 × 2 fiber coupler. The generated interference light is received by the spectrometer and focused on the line scan camera sensor after being unfolded by wavelength through the grating. The result is an interference spectrum carrying the displacement information of the structure to be measured. The Fast Fourier Transform (FFT) of the measured spectral interferogram provides the optical path difference of the reference light and measurement light, thus enabling the calculation of the difference in displacement between the reference and measuring arms.

In the optical coherence-based displacement-type weight sensing system, the interference spectra are recorded as data for the self-coherent intensities of reference light $I_{r}(\lambda_{i},t)$ and measuring light $I_{m}(\lambda_{i},t)$. The collected interference intensity $I(\lambda_{i},t)$ can be expressed as follows [18]:

(1) $$I(\lambda_{i},t)=I_{r}(\lambda_{i},t)+I_{m}(\lambda_{i},t)+2\sqrt{I_{r}(\lambda_{i},t)I_{m}(\lambda_{i},t)}\cos\left[\Delta \phi (\lambda_{i},t)\right]$$

(2) $$\Delta \phi (\lambda_{i},t)=\phi_{r}(\lambda_{i},t)-\phi_{m}(\lambda_{i},t)=\frac{4\pi n\Delta z(t)}{\lambda_{i}}$$

where $\Delta \phi (\lambda_{i},t)$ is the phase difference due to the displacement difference Δz(t) between the reference arm and measuring arm at time t when the wavelength is $\lambda_{i}$. Furthermore, $\lambda_{i}$ is the wavelength corresponding to the *i*th pixel of the interference spectrum acquired by the camera; t denotes time; and n denotes the refractive index of light in the propagation medium. Here, it is assumed that the value of the refractive index n=1, considering that only the surface layer of the elastomer material reflects light. 

If the material of the sample arm and reference arm is constant, the first two items of Equation (1) do not change, and the third term is the interference term carrying the structural information of the moving object. Therefore, we can ignore the first two items, and Equation (1) can be simplified as:

(3) $$I(\lambda_{i},t)=2\sqrt{I_{r}(\lambda_{i},t)I_{m}(\lambda_{i},t)}\cos\left[\frac{4\pi n\Delta z(t)}{\lambda_{i}}\right]$$

From Equation (3), it can be seen that the intensity of the interference signal is closely related to the phase difference, and the phase is determined by the optical path difference. Thus, the phase difference relationship between the minimum and maximum wavelengths can be obtained as:

(4) $$\Delta \phi (\lambda ,t)=\phi (\lambda_{1},t)-\phi (\lambda_{2},t)=\frac{4\pi n\Delta z(t)}{\lambda_{1}}-\frac{4\pi n\Delta z(t)}{\lambda_{2}}=\frac{4\pi n\Delta z(t)(\lambda_{2}-\lambda_{1})}{\lambda_{1}\lambda_{2}}$$

where $\lambda_{1}$ is the minimum wavelength and $\lambda_{2}$ is the maximum wavelength. According to the above equation, the number of interference signal cycles *N*(*t*) acquired by the CCD camera can be obtained as:

(5) $$N(t)=\frac{\Delta \phi (\lambda ,t)}{2\pi }=\frac{2n\Delta z(t)(\lambda_{2}-\lambda_{1})}{\lambda_{1}\lambda_{2}}$$

Hence, the displacement difference Δz(t) can be expressed as:

(6) $$\Delta z(t)=\frac{\lambda_{1}\lambda_{2}N(t)}{2n(\lambda_{2}-\lambda_{1})}$$

After calibrating the sensor through experiments, the relationship between the input weight m and output displacement difference Δz of the weight sensor can be obtained. Then, the weight signal and optical signal of the optical coherence-based displacement-type weight sensing model can be transformed by the following formula:

(7) $$m=\frac{1}{k}\Delta z(t)$$

where k is the sensitivity of the weight-sensing model obtained during the calibration process. Then, the broadband optical coherence-based displacement-type weighing sensing model can realize the conversion between weight signal and optical signal according to:

(8) $$m=\frac{1}{k}\frac{\lambda_{1}\lambda_{2}N(t)}{2n(\lambda_{2}-\lambda_{1})}$$

According to Equation (5), it can be seen that the number of cycles at different moments is also different, through the FFT can be extracted from the interference signal collected by the CCD camera so as to obtain the number of cycles of the interference signal intensity change of each line at that moment, and then according to Equation (6) can be obtained from the surface position of the object to be measured at each moment of the displacement difference Δz(t). After the calibration of the sensor, the relationship between the input and output can be obtained by the formula, and finally, according to Equation (7), the mass of the weight will be converted into the displacement difference between the reference arm and the measuring arm to realize the weighing sensing.

Our team has investigated the static characterization of the displacement-type weight sensor based on the optical coherence principle. The proposed weight sensor demonstrates good static properties and stability, a sensitivity of 146.814 μm/kg, a theoretical weight resolution of 0.004 g, and a nonlinearity error of 0.034% [13].

## 3. Dynamic Simulation Analysis of Weight Sensors 

To elucidate the dynamic characteristics of the weight sensor, a finite element simulation was conducted using ANSYS. A modal analysis was employed to identify the sensor’s natural frequencies. Harmonic response analysis and transient dynamic analysis were then utilized to simulate the frequency and time domain response characteristics of the elastomer, laying a robust theoretical groundwork for subsequent experimental testing

### 3.1. Modal Analysis

**Figure 4:**
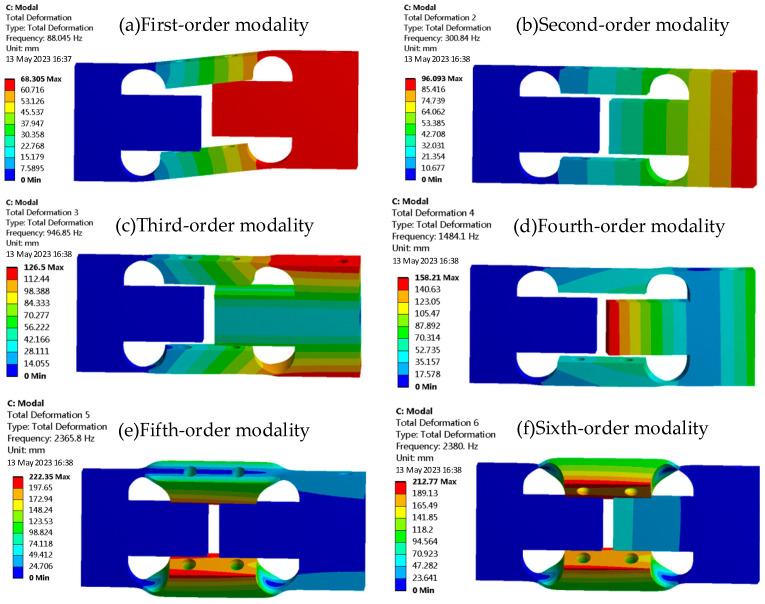
Mode shapes from modal analysis of the weight sensor.

Modal analysis offers insights into the intrinsic frequency and modal shape of the weight sensor, setting the stage for dynamic simulation analysis. Natural frequencies serve as the primary criteria for delineating the dynamic testing range of the weight sensor, with the primary frequency being especially pivotal among the structural natural frequencies. Based on the developed finite element model, a modal analysis is executed, revealing the first six mode shapes of the sensor, as illustrated in Figure 4. Their associated natural frequencies are detailed in Table 1.

During actual loading, the elastomer is mainly subjected to the longitudinal load. Combining the mode shapes, it is evident that the first-order natural frequency is the most influential. Therefore, the subsequent harmonic response analysis should include the first-order natural frequency, and the calculation frequency range can be set from 0 to 200 Hz.

### 3.2. Harmonic Response Analysis

**Figure 5:**
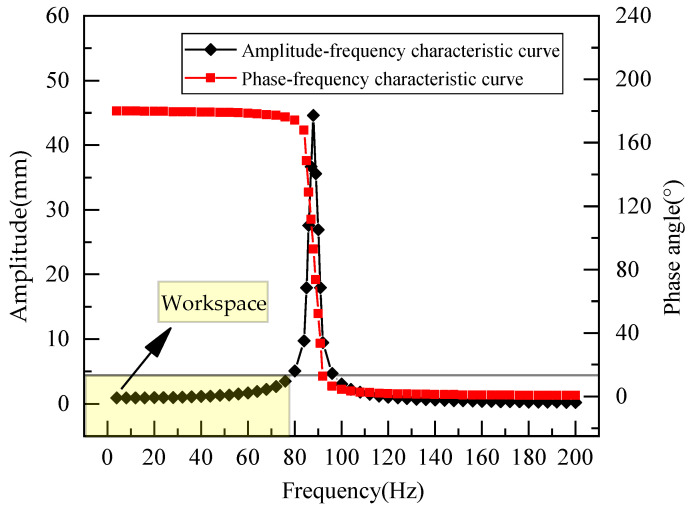
Simulation curve of frequency domain response characteristics.

A harmonic response analysis not only considers the properties of the material but also the impact of harmonic loads at different frequencies. Hence, it corresponds to an accurate method for analyzing the resonance frequency of the sensor, thereby determining the operating bandwidth. Using ANSYS, a load of $F_{y}=-58.729\;N$ was applied to the loading end. Based on the modal analysis results, the first-order natural frequency was considered as the primary influencing factor. Therefore, the frequency range was set from 0 to 200 Hz. The resulting frequency domain response characteristic curve is shown in Figure 5.

In Figure 5, a noticeable amplitude increase in the weight sensor is evident when a load is applied at 88 Hz. This observation aligns with the results from the modal analysis, suggesting resonance at this frequency. This type of resonance can significantly compromise the sensor’s capacity to weigh accurately. However, the amplitude–frequency curve remains fairly consistent within the frequency range of 0–78 Hz. The phase frequency curve shows a phase angle change that does not surpass 10° within this interval. Based on the frequency–response performance index, the frequency range where amplitude error remains within ±10% is considered the operating bandwidth [19]. Consequently, the region highlighted in yellow on the graph, representing the frequency range of 0–78 Hz, is determined as the operational range for the weight sensor, where it functions without distortion.

In Figure 5, the weight sensor displays a pronounced amplitude increase when exposed to a load at 88 Hz. This finding aligns with the modal analysis outcomes, suggesting a resonance at this specific frequency. This type of resonance can adversely affect the sensor’s accuracy in weighing. Conversely, the amplitude–frequency curve maintains a stable profile within the 0–78 Hz frequency range. Within this spectrum, the phase angle variation in the phase frequency curve stays under 10°. As stipulated by the frequency–response performance index, the frequency interval where the amplitude discrepancy remains below ±10% is designated as the operating bandwidth [19]. Hence, the section marked in yellow on the diagram, representing the 0–78 Hz frequency range, is identified as the weight sensor’s operational domain, where it functions without any distortion.

### 3.3. Transient Dynamic Analysis

Transient dynamic analysis directly reflects the dynamic performance of the sensor under time-varying loads. To simulate the subsequent negative step response experiment, in this study, the load provided by Equations (5) and (9), is applied to the elastomer, building upon the modal analysis. Considering that the sensor’s dynamic performance is better when used at loads below 50% of the rated load and with the laboratory’s gravity acceleration (26.08° N, 119.30° E) g = 9.788 m/s^2^, a dynamic analysis was performed on the elastomer with a 2 kg load, i.e.,

(9) $$P(t)=\left\{\begin{array}{l} F,\;\;t\leq 0.1\\ 0,\;\;\;0.1<t<2 \end{array}\right.$$

**Figure 6:**
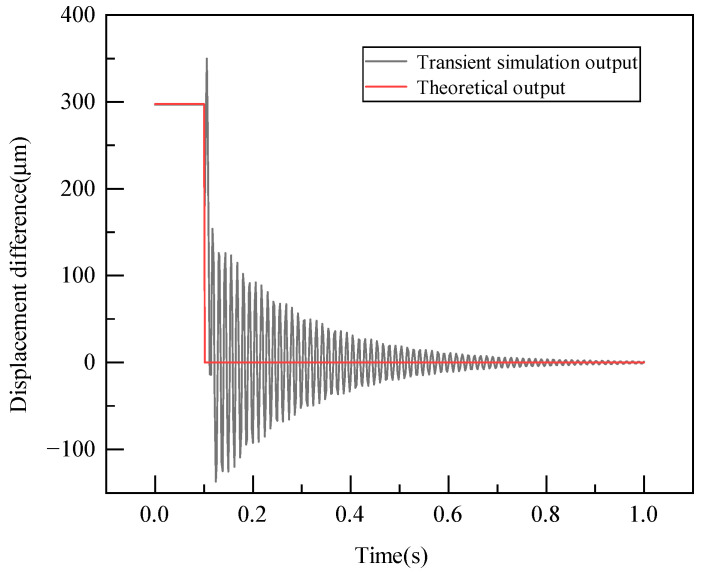
Simulation curve of negative step response of the weight sensor.

After conducting the transient dynamic analysis, the *Y*-direction displacement data at the central nodes of the measuring and reference arms were obtained. The difference in these displacements is then determined, leading to the negative step response simulation curve depicted in Figure 6. Following the swift load release, the transient phase requires a certain duration to complete. Hence, the initial displacement differential between the measuring and reference arms shows fluctuations. These fluctuations diminish quickly once the load is fully released and stabilize as time progresses. Using the time domain dynamic performance indicators [19], the sensor’s characteristics exhibited a rise time $t_{r}$ of 0.006 s, a peak time $t_{p}$ of 0.008 s, a response time $t_{s}$ of 0.43 s, and an overshoot σ of 51.74%.

## 4. Dynamic Characterization of Optical Coherence-Based Displacement-Type of Weight Sensing Principle

Finite element simulation analysis can only evaluate the dynamic performance of the sensor theoretically. In practical applications, there are complex operating conditions and influencing factors. Therefore, it is necessary to further test the weight sensor using experimental methods.

### 4.1. Dynamic Testing Experiment

#### 4.1.1. Experimental Method

**Figure 7:**
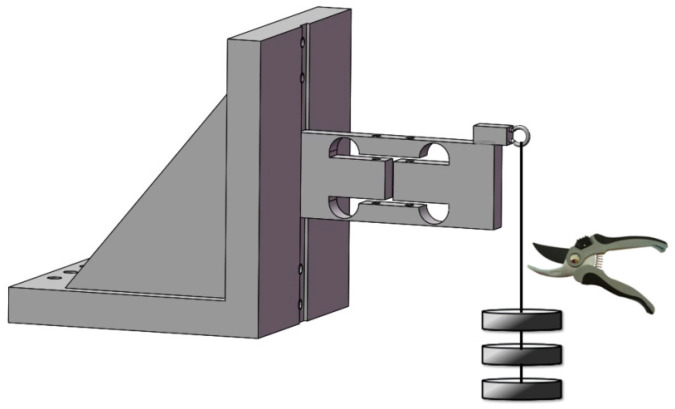
Schematic diagram of dynamic calibration principle of weight sensor.

Given that the step response method is straightforward to execute, offers strong repeatability, and excels in low-frequency characteristic assessments, and given that the step load is the most challenging input signal for sensors, it can be considered the most stringent test for evaluating dynamic performance [20]. Hence, in this study, this approach was selected for assessing dynamic performance, with the detailed principles depicted in Figure 7.

#### 4.1.2. Experimental Design and Platform Setup

**Figure 8:**
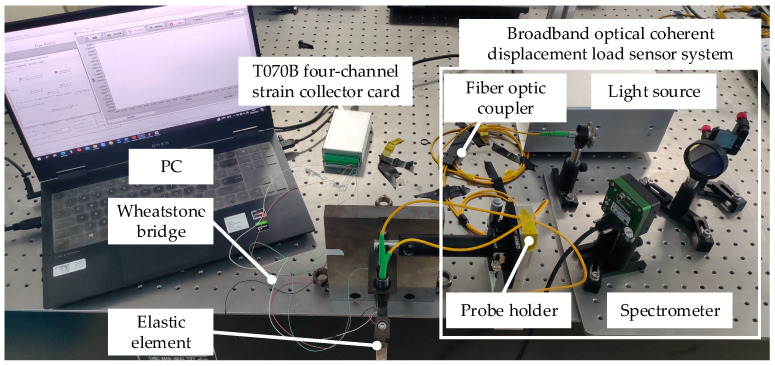
Setup of experimental platform to determine dynamic characteristics.

In this study, strain sensing and optical coherence-based displacement-type weight sensors were evaluated concurrently to contrast the dynamic response capabilities inherent in different weight sensor principles. Figure 8 illustrates the experimental platform designed for assessing the dynamic characteristics of these weight sensors. Within this framework, a high-precision T070B four-channel strain acquisition card (with a resolution of 24 bits and response frequency of up to 10 kHz) was employed for data collection from the strain sensors. For evaluating the optical coherence-based displacement-type weight sensor, a measurement system grounded on the principle outlined in Section 2 was constructed.

The broadband light source is an EXS210006-02 SLD superluminescent light-emitting diode with specifications of (841.6 ± 46.6) nm from EXALOS, which can emit broadband light with a center wavelength of 841.6 nm and bandwidth of 93.2 nm. The fiber optic coupler (50/50, 850 nm) uses 2 × 2 single-mode fiber for optical signal transmission. Additionally, the CCD camera is a high-speed OctPlus line-array camera with a maximum scanning line frequency of 250 kHz and a pixel size of 10 × 20, which is produced by TELEDYNE e2V and can quickly and efficiently collect the interference fringe pictures to ensure the high-frequency working ability of the load cell. The wavelength range ($\lambda_{1}-\lambda_{2}$) of the spectrometer constructed in this study was (810.81–872.43) nm after calibration. A reflective grating with dimensions of 25.4 mm × 25.4 mm was selected with a grating constant of 1/1800 nm and a diffraction angle of 20°. The focal length of the column lens was 75 mm, and the spectral resolution of the spectrometer was 0.070 nm. The line scan camera’s exposure time was set to 40 μs, and the spectrometer’s sampling rate was set to 10 kHz. With these parameters, the optical coherence-based displacement-type measurement system’s sampling time was 0.002 s, translating to a sampling frequency of 500 Hz. Based on the finite element analysis results discussed earlier, this sampling frequency is adequate for the intended dynamic performance tests.

#### 4.1.3. Experimental Procedure

**Figure 9:**
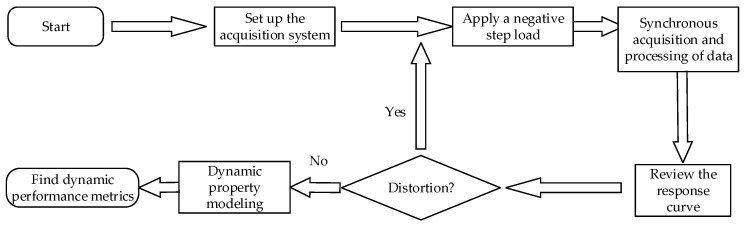
Steps for dynamic characteristics testing of weight sensors.

The procedure for testing dynamic characteristics is depicted in Figure 9. Upon assembling the experimental platform, a 0.5 kg weight was suspended from the elastomer’s pull ring, allowing the string to dangle freely. Data acquisition for the strain sensor and optical coherence-based sensor commenced once the string achieved stability. Subsequently, the string was swiftly severed using scissors, taking care to minimize any contact force during this action. Data collection concluded when the elastomer reverted to its original position. Following this, the response curves were scrutinized for any anomalies, and if distortions were detected, the test was rerun.

To ascertain consistency, this procedure was replicated thrice, with a new test initiated every 5 min. After completing the dynamic tests for the 0.5 kg weight, identical steps were executed using weights of 1.0 kg and 2.0 kg, culminating in nine comprehensive sets of experimental data.

### 4.2. Analysis of Experimental Results of Dynamic Tests

#### 4.2.1. Comparison Analysis of Different Sensing Principles

**Figure 10:**
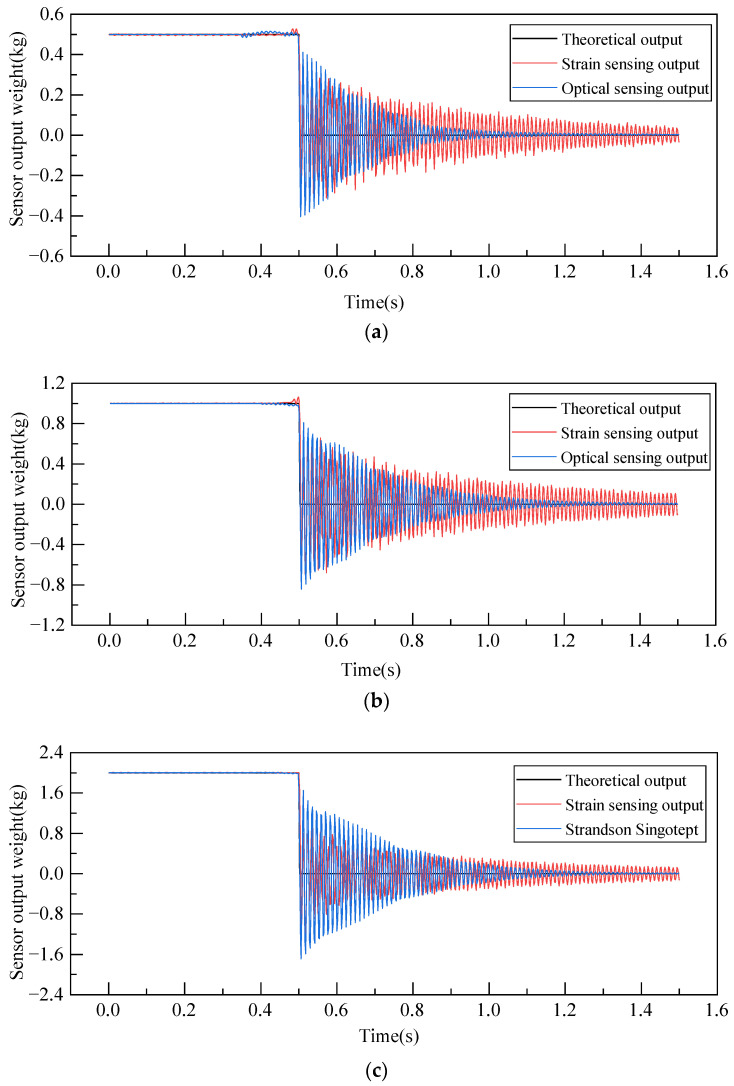
Sensor output comparison of different sensing principles. (**a**) Output comparison under 0.5 kg load. (**b**) Output comparison under 1.0 kg load. (**c**) Output comparison under 2.0 kg load.

For the strain sensor and optical coherence-based displacement-type weight sensor, the average negative step response outputs from the three dynamic characteristic tests are depicted in Figure 10a–c. As shown in Figure 10, when contrasting the strain sensor with the optical coherence-based displacement-type weight sensor, it is evident that, across varying loads, the latter tends to reach stabilization more swiftly. Once stabilized, the readings from both sensors align closely with theoretical expectations. This rapid stabilization in the optical coherence-based displacement-type weight sensor can be attributed to its non-contact sensing approach, which directly evaluates the elastomer, sidestepping the multi-layered transmission mechanism inherent in the strain sensor. Consequently, it can adjust and stabilize more rapidly. 

Based on the time domain dynamic performance indices, the dynamic response indices of two different weight sensing principles are shown in Table 2. From the table, it can be observed that as the load increases, there is no clear trend for rise time $t_{r}$, peak time $t_{p}$, overshoot σ, and response time $t_{s}$ (of increasing or decreasing), indicating that different loads do not significantly affect the dynamic response performance of weight sensors. After comparing the average values of the response indices, it can be determined that $t_{r}$ and $t_{p}$ of strain sensing are slightly lower than those of the optical coherence-based sensing. However, $t_{r}$ and $t_{p}$ are less than 0.01 s for both sensing principles, indicating that both sensing principles are sensitive and can respond rapidly under load. Regarding overshoot σ, the optical coherence-based displacement-type weight sensor can have a slightly higher overshoot than the strain sensor, but the overshoots of both types of sensors are below 85%. However, the response time $t_{s}$ of strain sensing far exceeds that of the optical coherence-based displacement-type weight sensing by a factor exceeding two. The comprehensive comparison suggests that optical coherence-based displacement-type weight sensing has significant advantages over strain sensing. It is more sensitive, responds faster, and has a shorter response time. Hence, the following text will analyze the optical coherence-based displacement-type weight sensing.

#### 4.2.2. Comparative Analysis of Experimental Testing and Simulations

**Figure 11:**
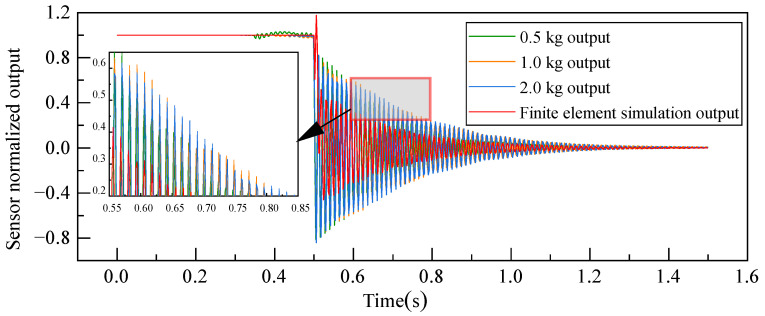
Comparison of step responses between dynamic experiment and finite element simulation.

In Figure 11, the findings from the optical coherence-based displacement-type weight sensing, derived from three separate measurements under varied loads, are normalized and compared against transient dynamic simulation outcomes. A discernible consistency in the dynamic response curves of the weight sensors can be observed across different load scenarios. This implies that the dynamic behavior of the sensors remains largely unchanged, irrespective of the magnitude of the load applied. Furthermore, the pattern of variations seen in the experimental sensor output resonates closely with those from the transient dynamic simulations. However, the experimental readings tend to register slightly higher than those projected by the finite element simulations. This discrepancy can be attributed to the simulation’s exclusive focus on the elastomer, leading to a degree of oversimplification and not accounting for external factors.

### 4.3. Dynamic Characteristics Modeling and Compensation

As analyzed in the preceding sections, varying loads do not significantly impact the dynamic response. Therefore, the acquired weight sensor data with an applied weight of 1.0 kg were subjected to smoothing, and the sensor’s transfer function was established using system identification, as follows:

(10) $$G(s)=\frac{308.4s+280300}{s^{2}+14.25s+281200}$$

**Figure 12:**
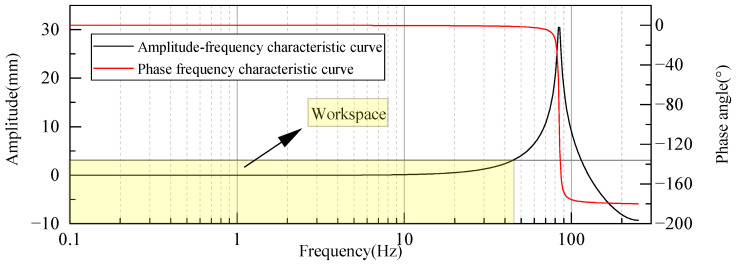
Frequency domain response curves obtained via system identification.

Based on the transfer function, the frequency domain dynamic response curves are obtained, as shown in Figure 12. From the figure, it can be observed that the sensor’s natural frequency is 85 Hz, which closely aligns with the finite element simulation results. The operating bandwidth is 0–44.53 Hz, showing some deviation when compared to the earlier harmonic response analysis due to the idealized simplification of the finite element model. In practical weight measurements, the sensor operates in complex environments affected by various factors.

From Figure 12, it is evident that the dynamic response of the designed optical fiber weight sensor is suboptimal, characterized by a significant overshoot and prolonged settling time. To address these issues, in this study, the pole-zero placement method [21] was utilized. This method dynamically compensates for the weight sensor’s behavior, effectively reducing overshoot and response time while expanding its operational bandwidth.

Based on the dynamic modeling results, the transfer function in this study is a second-order model, represented as follows:

(11) $$H(s)=\frac{b_{1}s+b_{2}}{s^{2}+a_{1}s+a_{2}}$$

where $a_{1}$ and $a_{2}$ represent the input parameters of the second-order sensor system, respectively. Without adjusting the zero, the following second-order homogeneous compensation model can be derived by replacing model poles as follows:

(12) $$H_{c}(s)=\frac{(s^{2}+a_{1}s+a_{2})\omega_{n}^{2}}{(s^{2}+2\xi \omega_{n}+\omega_{n}^{2})a_{2}}$$

where, ξ denotes the damping ratio; $\omega_{n}$ denotes the natural frequency. To satisfy the application requirements of the weight sensor, they are set to ξ=0.7 and $\omega_{n}=200$. Then, the desired compensator model can be derived as:

(13) $$H_{c}(s)=\frac{0.1424s^{2}+2.0092s+40000}{s^{2}+280s+40000}$$

**Figure 13:**
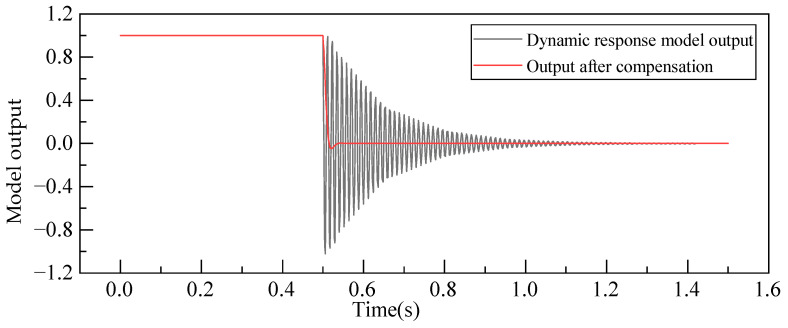
Comparison of model outputs before and after dynamic compensation.

A comparison of weight sensor outputs before and after compensation yields the dynamic response curves illustrated in Figure 13, and the time domain dynamic performance indices of weighing sensors before and after compensation are shown in Table 3. It can be observed that after dynamic compensation, the weight sensor’s overshoot is reduced to 4.72%, a significant improvement when compared to the initial state. Simultaneously, the response speed significantly increases, with a shortened response time of 0.0132 s, effectively meeting the rapid weight measurement requirements of the weight sensor. Although the rise time and peak time experience some increase, they do not adversely affect the overall improvement in dynamic weighing performance.

**Figure 14:**
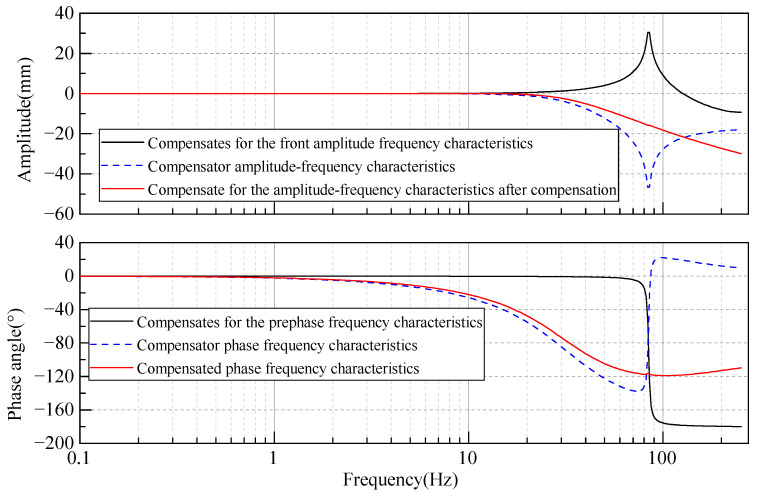
Comparison of frequency–response curves before and after weight sensor compensation.

To further observe the enhancement of the weight sensor’s frequency domain characteristics, a comparison of the frequency–response curves is presented in Figure 14. This graph clearly shows that, after compensation, the weight sensor no longer exhibits resonance points, and its operating bandwidth is significantly improved, indicating significantly enhanced interference resistance.

## 5. Conclusions

This study introduced an optical coherence-based displacement-type weight sensor and delved into its dynamic characteristics. The dynamic performance of the weight sensor was examined via finite element simulations. A model analysis identified the sensor’s inherent frequencies, and both harmonic response and transient dynamic analyses were used to explore its dynamic response in frequency and time domains. From this, the theoretical indices for the sensor’s dynamic performance were derived. Following this, dynamic performance tests compared weight sensors based on two distinct sensing principles, employing the negative step response technique. Comparative testing revealed that while both sensing modalities demonstrate prompt response dynamics, the strain sensing approach exhibited a response time over double that of the optical coherence-based displacement-type weight sensor. This underscores the enhanced dynamic performance, swifter response rate, and reduced stabilization period inherent to non-contact optical coherence-based sensing.

To rectify the sensor’s limitations, such as high overshoot and extended response duration, we employed the pole-zero placement technique. This modification reduced the overshoot to a mere 4.72% and shortened the response time to 0.0132 s, enhancing the sensor’s response agility and expanding its operational frequency bandwidth.

## Figures and Tables

**Table 1:** Natural frequencies and mode shapes of the elastomer.

Order	Natural Frequency/Hz	Mode Shape
1	88.045	Vibration along the *Y*-axis for the XOY plane
2	300.84	Vibration along the *Z*-axis for the XOZ plane
3	946.85	Torsional vibration around the *X*-axis
4	1484.1	Alternating vibration along the *Z*-axis for the XOZ plane
5	2365.8	Opposite-direction vibration of the upper and lower beams on the XOY plane
6	2380.0	Same-direction vibration of the upper and lower beams on the XOY plane

**Table 2:** Time domain dynamic performance indices of different weight sensing principles.

Load/kg	Sensing Principle	Rising Time *t_r_*/s	Peak Time *t_p_*/s	Response Time *t_s_*/s	Overshoot σ/%
0.5	Optical sensing	0.006	0.008	0.458	82.20
	Strain sensing	0.003	0.006	1.270	63.60
1.0	Optical sensing	0.007	0.01	0.592	84.28
	Strain sensing	0.004	0.006	1.414	72.42
2.0	Optical sensing	0.007	0.01	0.606	84.23
	Strain sensing	0.004	0.006	1.160	60.70
Average	Optical sensing	0.007	0.009	0.552	83.57
	Strain sensing	0.004	0.006	1.281	65.57

**Table 3:** Time domain dynamic performance indices of weighing sensor before and after compensation.

Load/kg	Weighing Sensor	Rising Time *t_r_*/s	Peak Time *t_p_*/s	Response Time *t_s_*/s	Overshoot σ/%
1.0	Before compensation	0.007	0.010	0.5920	84.28
	After compensation	0.010	0.016	0.5788	4.72

## Data Availability

No data were used for the research described in the article.
